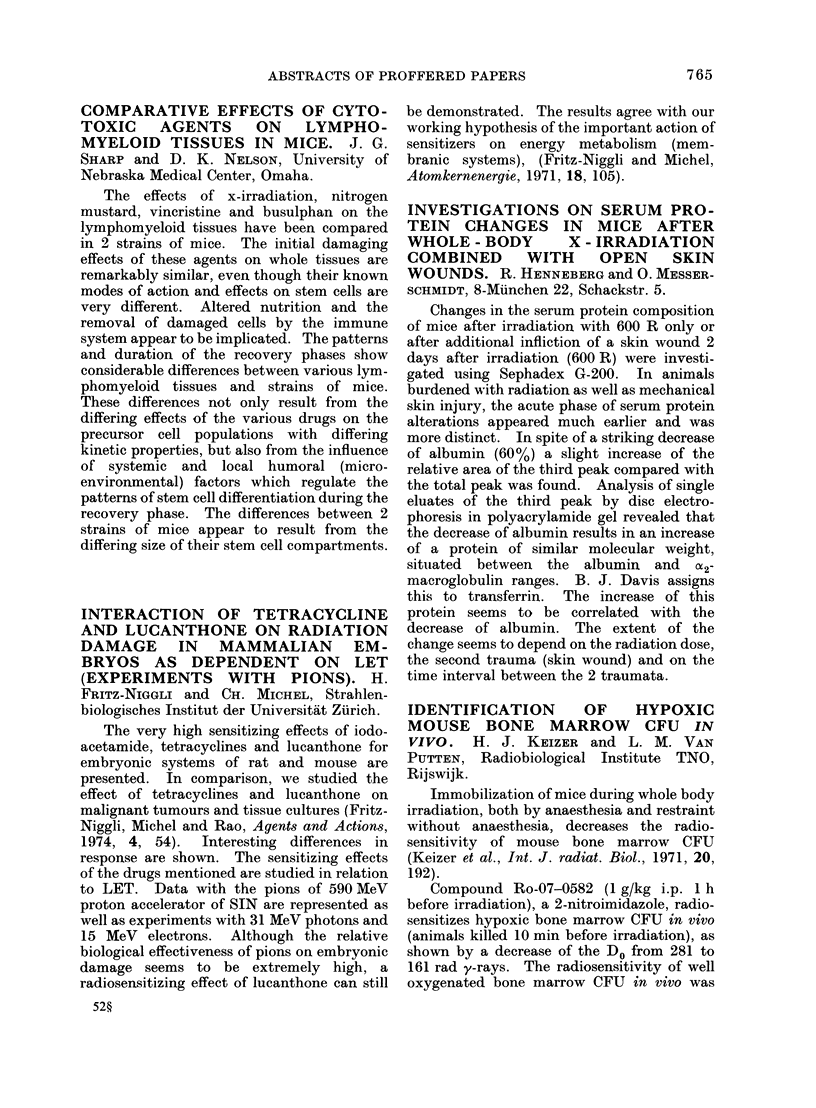# Proceedings: Interaction of tetracycline and lucanthone on radiation damage in mammalian embryos as dependent on LET (experiments with pions).

**DOI:** 10.1038/bjc.1975.335

**Published:** 1975-12

**Authors:** H. Fritz-Niggli, C. Michel


**INTERACTION OF TETRACYCLINE AND LUCANTHONE ON RADIATION DAMAGE IN MAMMALIAN EMBRYOS AS DEPENDENT ON LET (EXPERIMENTS WITH PIONS).** H. FRITZ-NIGGLI and CH. MICHEL, Strahlenbiologisches Institut der Universität Zürich.

The very high sensitizing effects of iodoacetamide, tetracyclines and lucanthone for embryonic systems of rat and mouse are presented. In comparison, we studied the effect of tetracyclines and lucanthone on malignant tumours and tissue cultures (Fritz-Niggli, Michel and Rao, *Agents and Actions*, 1974, **4**, 54). Interesting differences in response are shown. The sensitizing effects of the drugs mentioned are studied in relation to LET. Data with the pions of 590 MeV proton accelerator of SIN are represented as well as experiments with 31 MeV photons and 15 MeV electrons. Although the relative biological effectiveness of pions on embryonic damage seems to be extremely high, a radiosensitizing effect of lucanthone can still be demonstrated. The results agree with our working hypothesis of the important action of sensitizers on energy metabolism (membranic systems), (Fritz-Niggli and Michel, *Atomkernenergie*, 1971, **18**, 105).